# Urinary oxytocin levels in children meeting a Hospital Dog^®^

**DOI:** 10.1186/s12906-025-05076-6

**Published:** 2025-09-08

**Authors:** A. Risberg, A. Larsson, U. Bodén, A. Edner

**Affiliations:** 1 Department of Health, Education and Technology, Lulea University of Technology, Lulea, S-971 87 Sweden; 2 Department Medical Sciences, Clinical Chemistry, Academic Hospital, Uppsala, S- 75185 Sweden; 3 Karolinska University Hospital, Huddinge, S-141 57 Sweden; 4 Department of Women´s and Children´s Health, Uppsala University, Uppsala, S-751 85 Sweden

**Keywords:** Hospital Dog^®^, Dog therapy, Children, Oxytocin, Urine oxytocin

## Abstract

**Abstract:**

There has been growing interest in animal-assisted therapy (AAT) in recent decades due to increasing reports indicating its health benefits for adult patients. These benefits are partly attributed to changes, usually increased levels of the neuropeptide oxytocin.

**Aim:**

To investigate changes in oxytocin levels in hospitalized children meeting a certified Hospital Dog^®^.

**Method:**

Urine samples were collected between 25/02/2016 and 24/05/2017 from 35 hospitalized children (3–17 years) before and after each participant had a session with the Hospital Dog^®^. Oxytocin levels were analysed with an acetylcholinesterase (AChE) competitive enzyme-linked immunosorbent assay (ELISA). Creatinine levels were measured to determine the subject’s fluid intake and then divided by the hormonal concentration (uOT pg/ml).

**Results:**

The mean level of uOT was 186.0 ± 236.7 (51.5–1349.5) pg/ml before and 137.3 ± 121.5 (30.7–591.3) pg/ml after the dog session (*p* = 0.010).

**Conclusion:**

Decreased levels of uOT were recorded during the study in which hospitalized children met a Hospital Dog^®^. The decreased OT levels are potentially the result of the intense activity the subject experienced with the dog during the interaction.

## Introduction

In recent years, substantial medical progress has been made in improving children’s overall well-being, significantly reducing child mortality. One of the consequences of this has led to an increasing number of children living with chronic illness and/or long-standing functional disabilities, often involving numerous hospital visits, including extended stays [1]. Regardless of one’s age, being admitted to the hospital is frequently associated with much anxiety and, for some, an emotionally traumatic experience. Children are particularly vulnerable to the negative effects of being ill, but in combination with hospitalizations, this can result in heightened stress levels for both the child and their family [2]. For anyone, but especially for a child living with chronic disease, the long-term impact of chronic disease may eventually lead to fatigue, reduced mobility, and loss of bodily functions, placing the body under continuous duress [3]. In addition to experiencing stress caused by physical suffering from the disease, a child is exposed to other factors that negatively affect psychological well-being [4]. Examples of adverse factors include painful medical procedures, a foreign environment, feeling isolated, and not having their families close by. This has been confirmed to contribute to a child’s negative response to both the hospital stay and medical treatment [5].

Under stressful conditions, the child becomes more dependent on support from the family or relatives. A child’s coping mechanisms and emotional resources are not yet developed to manage the tremendous psychological and emotional stress they experience during a hospital stay [1]. Frequent or extended hospital stays are thought to negatively affect patients’ psychological state and increase the risk of long-term emotional and behavioral difficulties [6].

Complementary interventions such as clowns, music, film, and play therapy have become a major component of children’s healthcare today. These therapies are recognized as important factors in helping children in stressful hospital environments develop strong coping mechanisms. Game-like activities relieve anxiety but, above all, increase children’s ability to adapt. During play, the child can develop the ability to reason, think, and solve problems. Children who are good at playing also seem better at handling cognitively challenging situations. In addition, playing gives the child a sense of control over their life. However, seriously ill children are more likely to engage in repetitive, quiet, solitary play, which does not stimulate the abilities mentioned above in the same way [7].

Another type of complementary treatment that has become increasingly common is “animal-assisted therapy” (AAT), where the respective animal is often a specially trained dog [8]. Dr. Boris Levinson started the animal-assisted therapy movement. Levinson’s study indicated that adult patients became calmer when a dog was present at the sessions. Dr. Samuel Corson developed what he called pet-assisted therapy [9]. Most of the research on AAT has been carried out on the adult population, which has indicated that AAT therapy reduces pain, depression, and fatigue in patients with various types of medical conditions [10].

**Fig. 1:**
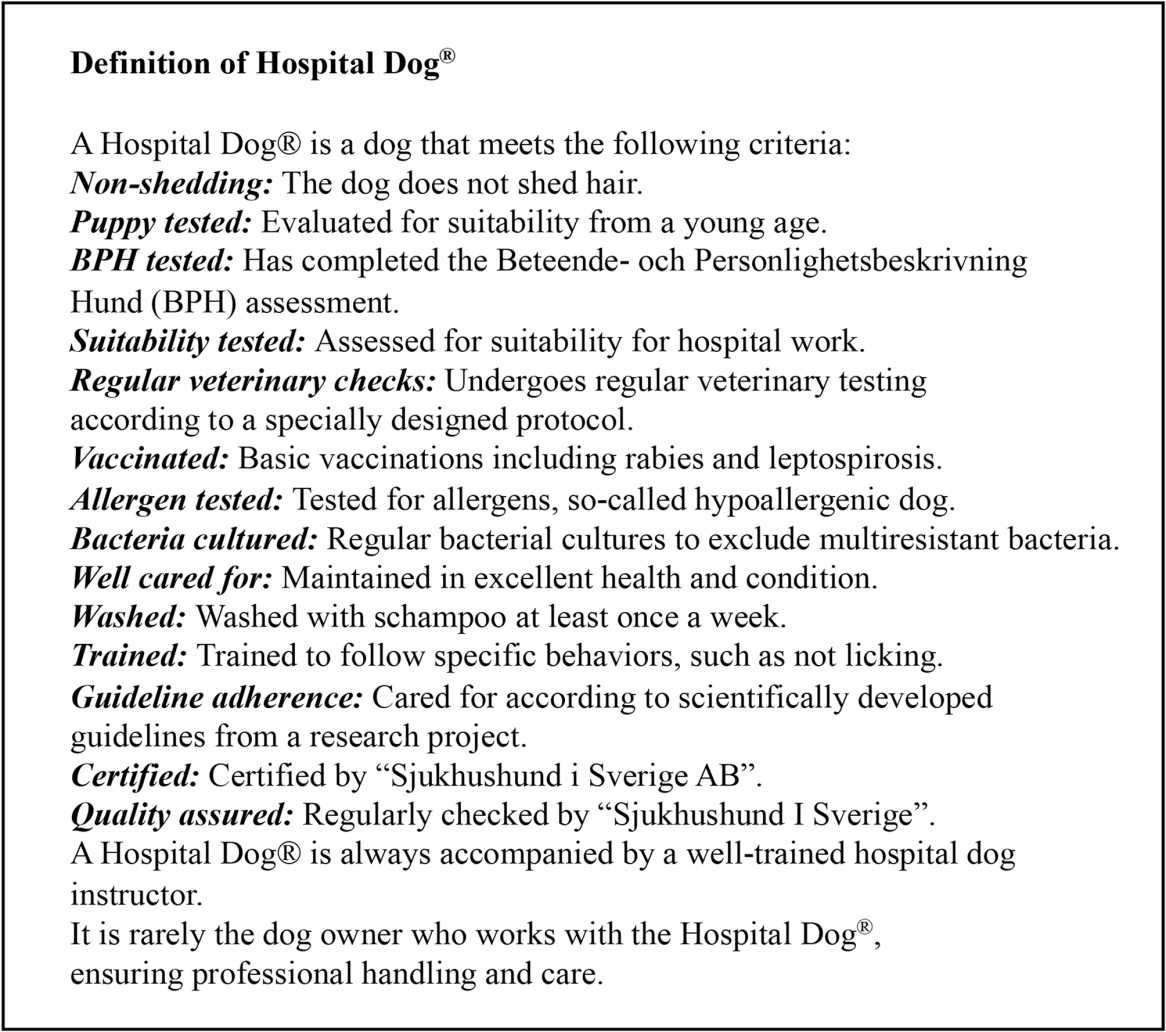
Definition of Hospital Dog^®^

Although not as many trials have been conducted in the paediatric population on dog therapy, beneficial effects have been documented. In a 2006 study, a pre- and postoperative questionnaire was used to show a significant reduction in postoperative pain and anxiety in children between 5 and 17 years of age. Based on interviews after the intervention, the authors explained the results by the dog’s ability to distract the child from the pain or situation and act as a soothing agent [11]. Another study in 2009 compared pain ratings between two paediatric hospitalized groups. One group was given a 15–20-minute session with a dog, while the control group was assigned to rest in silence for the same time. The study showed that the children who were allowed to interact with a dog had a pain reduction that was four times greater than that of the children who rested in silence [12]. A study conducted in a paediatric oncology department in Canada noted that 89% of children who received AAT experienced an improvement in independence and appetite, a decrease in fear and pain, and positive effects on various psychological and psychiatric conditions [13]. However, it has not been possible to let a dog, now named Hospital Dog^®^ (Definition see Fig. 1), work inside Swedish hospitals before scientific patient-safe guidelines were produced, of which this study is a part. This research has been necessary because of the strict guidelines in Sweden regarding health hygiene and allergen excretion, led to the development of the integrated medicine Hospital Dog^®^ [14].

Many studies have shown that hospitalized children are exposed to enormous stress due to both mental and physical strain. When humans respond to stress, the sympathetic nervous system is activated, leading to an increase in cardiac output and heart rate [15]. The locus coeruleus is centrally located in the pons of the brainstem and is involved in physiological responses such as attention, arousal, motivation, and cognitive function. During sympathetic activation, corticotrophin-releasing factor and vasopressin are released from the paraventricular nucleus (PVN). These two peptides also activate the hypothalamic-pituitary-adrenocortical axis (HPA), releasing adrenocorticotrophic hormone (ACTH) and stimulating cortisol secretion. Cortisol provides a constant energy supply for the body to deal with stress [16].

Oxytocin (OT) has many behavioural and physiological functions in the body, both centrally and peripherally [17]. Among other things, OT also includes nonpainful sensory stimuli such as skin-to-skin contact and warm bodily interaction [16].

Through the work of Kerstin Uvnäs-Moberg, it has been established that oxytocin lowers blood pressure, affects certain gastrointestinal hormones and spontaneous motor activity, increases the pain threshold, and reduces corticosterone secretion, which has a stress-relieving effect [16]. Stimulation of the alpha 2-adrenoceptor produces effects such as analgesia, anxiety reduction, sedation, hypotension, and a reduction in sympathetic activity [18].

Because many of the positive health benefits of Hospital Dog^®^ overlap with those of oxytocin, one would expect that stimulating OT release would have similar medical benefits. Earlier studies have confirmed that adults experience elevated blood oxytocin concentrations after they have spent time interacting with a dog [19]. The aim of this study was to investigate variations in children’s OT levels by collecting urine samples from each subject before and after playing with a specially certified Hospital Dog^®^.

## Materials and methods

### Study design

The study was set up as an intervention trial, each subject spending time interacting with a certified Hospital Dog^®^. Each participant’s urine oxytocin level was collected before and after the dog visit and later examined to determine whether there was a change in the OT release.

### Study population

Fifty patients receiving inpatient care at a children’s hospital were screened for inclusion in the study. However, based on the exclusion criteria and insufficient urine samples, only thirty-five children aged between 3 and 17 years were included. The subjects selected for this study were receiving inpatient care at a 25-bed children’s neurological, orthopedic, surgical, or urological ward at the Academic Children’s Hospital in Uppsala, Sweden (Table 1). The trial was registered at clinicaltrial.gov 03/05/2024, NCT06414876.

**Table 1:** Demographic data and diagnosis (*n*=50)

Sex	Number	Mean	Median
Boys/Girls	24/26		
Age	11.5		12
3–6	9		
7–9	4		
10–12	14		
13–15	17		
16–18	6		
Diagnosis			
Neuro-oncology	7		
Brain damage	4		
Epilepsy/Other neurological condition	2		
Minor surgical procedure	11		
Medium surgical procedure	16		
Major surgical procedure	2		
Trauma	2		

### Procedure

The study started 25/02/2016 and the study ended 24/05/2017. Each morning that the intervention was to occur, a random administrator was identified to assign a sequential number to the inpatient children who qualified for the study. The process was conducted as follows: Child number one and their parents were verbally asked if they were interested in participating in the trial and, at the same time, given a copy of the informed consent to read. If they chose not to participate, the next child and their parents were asked to participate. Those who chose to participate signed the informed consent form after all questions were answered and were given a personal copy of the signed document. Children with widespread eczema or ulcers, diarrhoea, immunosuppression, multidrug-resistant bacteria, or short bowel syndrome with a central venous catheter for parenteral nutrition were excluded.

A certified Hospital Dog^®^ (a six-year-old labradoodle) was used for the intervention, and the dog was experienced and well trained – see Definition of Hospital Dog^®^. The dog had been working with children and young people for many years, first at a children’s hospice and then at a children’s hospital. The instructor of the Hospital Dog^®^ attended every meeting with the Hospital Dog^®^ and each subject.

The Hospital Dog^®^ met one to three children at least 24 h apart per week. The visits primarily took place in the child’s hospital room. A few children who participated in the intervention were in double rooms, which meant moving to a single room for privacy during the dog visit. Before each patient’s visit, the dog was washed with a specially developed shampoo that reduced allergens and then blow-dried completely dry. During the meeting, when the dog was allowed to get on the child’s bed, an extra sheet was used between the dog’s and the child’s bedding. After the meeting, all bedding that the Hospital Dog^®^ had been in contact with was removed, and the room was thoroughly cleaned.

Each subject’s urine was collected in the morning before the dog meeting and at each child’s first bathroom visit after dog therapy. In those cases where the children had catheters, urine was collected from the catheter before and after the dog session. Each session began with a quiet activity where the child and the Hospital Dog^®^ got to know each other. For example, this could mean that the child was allowed to pet the hospital dog while she was lying next to the child in bed. Then, the child was asked to choose a livelier activity, such as letting the Hospital Dog^®^ pick up rings and put them on a cone and perform different tricks at the child’s command, such as lying down, rolling over, sitting, high five, playing dice or looking for dog treats in a puzzle. The session with the dog then ended by returning to a calmer activity similar to the one when the session started.

By measuring pre- and postintervention, each subject’s preurine levels became that child’s base value. As it was not possible to analyse the urine immediately after the session, the urine was stored in a freezer (−70 °C) until analysis.

### Determination of oxytocin

The level of OTs in the urine was determined using the acetylcholinesterase (AChE) competitive enzyme-linked immunosorbent assay (ELISA) and Cayman’s oxytocin ELISA kit. Measurements were performed in duplicate. The observed intra-assay and interassay coefficients were < 8% and 10%, respectively. The creatine levels in the urine were also measured to compensate for the varying fluid intakes of the research subjects.

The oxytocin concentration was then divided by the urinary creatinine concentration (uCreatinine). This ratio is hereafter referred to as “uOT”, which is the abbreviation for (urine OT), (pg/mg).

### Statistical methods and data management

Statistical analysis was performed using SPSS version 22.0 (IBM Corp., Armonk, NY, USA), and unless otherwise stated, all the data are presented as the mean ± standard deviation (SD). Continuous data were analysed with a t test. Statistical assessment of paired observations was performed using paired t tests. Quantitative, nonnormally distributed variables were logarithmically transformed before the t test. Values are presented as the median, range, and 95% confidence interval (CI) when appropriate to evaluate the relationship between uOT, age, and time with a dog. Pearson’s correlation method was used for the girl and boy groups throughout the trial. A 2-sided p value < 0.05 indicated a significant difference.

## Results and discussion

### Results

Urine samples were collected from 35 children. Two urine test tubes were removed from the freezer, and one tube was excluded, resulting in the analysis of 32 urine samples. Thirty-one urine samples were therefore included in the statistical analysis, 19 from girls and 12 from boys. One child’s urine samples were excluded because the samples collected before and after the dog encounter had oxytocin concentrations approximately ten times higher than those of the other samples, which the computer analysis labelled “out of range.” Thirty-one urine samples were therefore included in the statistical analysis, 19 from girls and 12 from boys. The mean age of the children whose urine was analysed was 12.3 ± 3.9 years (3–17 years). The mean time between the collection of the pre- [1] and post- [2] urine tests was 3 h and 36 min (1–11 h), respectively. The average time the children spent with the dog was 40 min (29–65 min).

In the urine samples collected before the dog meeting, the oxytocin concentration differed between the duplicates by an average of 10.1 pg/ml, compared to 11.0 pg/ml in the urine samples taken after the dog meeting. The mean value of the duplicates is used for future calculations. The mean oxytocin concentration before correction for creatinine (*n* = 31) taken before the dog encounter was 167.9 ± 157.2 (7.4–646.0) pg/ml, and it was 115.5 ± 112.3 (5.8-478.3) pg/ml after the dog encounter (see Table 2).

**Table 2:** uOT before and after meeting with the dog for the whole group, girls and boys. Values are expressed as Mean ±SD (range) and CI 95%

	Before Mean ± SD (range)	After Mean ± SD (range)
uOT all *n* = 31	186.0 ± 236.7 (51.5–1349.5)	137.3 ± 121.5 (30.7–591.3)
	pg/ml	pg/ml (*p* = 0.010)
uOT girls *n* = 19	191.7 ± 290.4 (51.5–1349.5)	139.4 ± 123.9 (35.4–591.3)
	pg/ml	pg/ml (n.s.)
uOT boys *n* = 12	176.9 ± 121.3 (53.9–411.0)	133.9 ± 123.0 (30.7–467.2)
	pg/ml	pg/ml (*p* = 0.007)

The mean uOT (*n* = 31) before the dog encounter was 186.0 ± 236.7 (51.5-1349.5) pg/ml, and it was 137.3 ± 121.5 (30.7-591.3) pg/ml after the dog encounter (*p* = 0.010). A statistically significant decrease in urinary oxytocin (uOT) in the boys’ group (*n* = 12) was also observed, from 176.9 ± 121.3 pg/ml before dog encounters to 133.9 ± 123.0 pg/ml after dog encounters (*p* = 0.007). However, no statistically significant change was observed in the girl group (*n* = 19).

No correlation between changes in urinary oxytocin and age or time with the dog was detected in the entire study group or among the girls’ and boys’ groups. This study revealed a statistically significant reduction in uOT in children after dog encounters. This result raises many questions in the main study, as 93% of the children who met the Hospital Dog^®^ indicated increased well-being in questionnaire responses. Increased well-being is associated with an increase in plasma OT in the adult population.

### Results and method discussion

Minimizing the child’s anxiety and traumatic experience of hospitalization through complementary therapy is currently a natural component of paediatric care. Dog therapy is a complementary treatment that has been shown to have positive health effects, such as stress and pain relief, in adults. Research conducted in the adult population has shown that it is possible to connect many of the positive effects from dogs to increased OT release, which in turn seems to redirect the autonomic nervous system in a parasympathetic direction. However, only a limited number of studies done at hospitals outside Sweden have shown this in a paediatric population, which is why this study tested whether an increase in OT could be detected in the urine of hospitalized children and adolescents allowed to spend time with the Hospital Dog^®^.

The urinary OT levels in this study were generally higher than those in studies such as Feldman, 2011, which analysed urinary OT. In studies in which OT was analysed in urine by ELISA, the results in healthy men and nonpregnant women had mean levels of 10 pg/ml [20] compared to the results in our study, which showed levels between 12 and 669 pg/ml and an average value of approximately 200 pg/ml. This high mean value and the large spread of the oxytocin concentration could have several explanations. However, further speculations are worth discussing, such as an unexplored hypothesis that children release OT more quickly and/or that their kidneys eliminate more OT via urine.

The lack of increase in the OT concentration in the children in the trial, as noted in the adult study, may be interrelated with children’s keen imagination, given that the children were informed before the first urine sample that they would reach the Hospital Dog^®^. The children’s excitement by simply thinking about the upcoming dog meeting could have resulted in a possible increase in OT levels. A control group could have shown the OT concentration in children and young people who did not have this expectation and experience.

If the connection that OT seems to have with the autonomic nervous system is correct, sympathetic activation means a reduced release of OT. Because each subject was assigned a combined session (first a quiet activity with the Hospital Dog^®^, subsequent livelier activity with the dog, ending with a calm activity with the dog), a sympathetic impulse may have led to a reduced OT release. If the study included only quiet activity alone and quiet activity with the Hospital Dog^®^, for example, only cuddling in bed, perhaps the OT levels would have risen.

Another explanation for the reduced OT concentration is the diurnal variation of the peptide. In a study by Forsling et al., 1998, plasma OT levels were analysed in 15 young men over a 24-hour period [21]. The study showed that the OT level was highest at 04:00 and lowest at 16:00 and 24:00 [21]. This means that the first urine sample was taken when OT levels were about to decline from their peak. The second urine sample was collected during the period when OT levels were at their lowest level compared to the study performed by Forsling et al. [21]. As we lacked normal range values for OT in the children whose urine was analysed, it is difficult to determine how much the diurnal variation impacts the results. As this study showed, slightly decreasing levels between the morning and afternoon could indicate a relative increase in relation to the diurnal variation in OT in children.

Urine sampling was used to avoid invasive methods in children. There is also limited knowledge about the metabolism and elimination of OT, which needs further study. According to Amico et al. [22], 1987, who performed a clearance study on oxytocin, only 1% of plasma OT is filtered into the urine. They also found that randomized collection of urine correlated poorly with plasma levels of OT [23].

One limitation is the variation of diurnal OT variation, as mentioned above and another limitation is that OT is not related to heart rate variability to understand whether the child is in a parasympathetic or sympathetic state – which explain if the child is calm or not. Desirable had been to have a matched control group, though it is ethically difficult to get approval in Sweden concerning doing research on sick children.

## Conclusion

Current evidence indicates that animal-assisted therapy/meeting a Hospital Dog^®^ has many advantages for both adults and children. Like all types of treatment, however, further studies with dog therapy before it can become an established therapeutic method are warranted. This study demonstrated a decrease in oxytocin levels in the urine of children who were allowed to spend time with a Hospital Dog^®^. In future trials, it is desirable to study children who only have calm contact with a Hospital Dog^®^ to determine whether this leads to higher levels of OT, as in adults, compared to the present study, in which the activity and calm parts of the dog were combined. Hence, it was difficult to determine the outcome. It would also be interesting to perform a comparison follow-up between OT in plasma, saliva, and urine in children to determine the elimination of OT and assess heart rate variation and cortisol as a control of performance levels to determine sympathetic and parasympathetic activity in children related to oxytocin.

## Data Availability

The dataset used and/or analysed during the current study available from the corresponding author on reasonable request.

## Abbreviations

AAT: Animal-assisted therapy
AChE: Acetylcholinesterase
ACTH: Adrenocorticotrophic hormone
ELISA: Enzyme-linked immunosorbent
HPA: Hypothalamic-pituitary-adrenocortical axis
PVN: Paraventricular nucleus
uOT: Urine Oxytocin
iCreatinine: Urinary creatinine concentration

## References

[1] Boyd JR, Hunsberger M. Chronically ill children coping with repeated hospitalizations: their perceptions and suggested interventions. J Pediatr Nurs. 1998;13:6–pp. 10.1016/S0882-5963(98)80021-3 9879169

[2] Kennedy C, Kools S, Kong SKF, et al. Behavioural, emotional and family functioning of hospitalized children in China and Hong Kong. Int Nurs Rev. 2004;51:34–46. 14764013 10.1111/j.1466-7657.2003.00204.x

[3] Rowe MA. The impact of internal and external resources on functional outcomes in chronic illness. Res Nurs Health. 1996;19(6):485–97. 8948402 10.1002/(SICI)1098-240X(199612)19:6<485::AID-NUR4>3.0.CO;2-K

[4] Rattray JE, Johnston M, Wildsmith JAW. Predictors of emotional outcomes of intensive care. Anaesthesia. 2005;60(11):1085–92. 16229693 10.1111/j.1365-2044.2005.04336.x

[5] Longhi E, Pickett N, Hargreaves DJ. Wellbeing and hospitalized children: can music help? Psychol Music. 2015;43(2):188–96.

[6] Rokach A, et al. Psychologicmotional and physical experiences of hospitalized children. Clin Case Rep Rev. 2016;2(4). Available: http://oatext.com/Psychological-emotional-and-physical-experiences-of-hospitalized-children.php.

[7] Gariepy N, Howe N. The therapeutic power of play: examining the play of young children with leukemia. Child Care Health Dev. 2003;29(6):523–37. 14616910 10.1046/j.1365-2214.2003.00372.x

[8] Willens JS. Animal-Assisted therapies are becoming more common. Pain Manag Nurs. 2013;14(4):183. 24315240 10.1016/j.pmn.2013.10.001

[9] Goddard AT, Gilmer MJ. The role and impact of animals with pediatric patients. Pediatr Nurs. 2015;41(2):8. 26292453

[10] Marcus DA. Complementary medicine in cancer care: adding a therapy dog to the team. Curr Pain Headache Rep. 2012;16(4):289–91. 22544640 10.1007/s11916-012-0264-0

[11] Sobo EJ, Eng B, Kassity-Krich N. Canine visitation (Pet) therapy: pilot data on decreases in child pain perception. J Holist Nurs. 2006;24(1):51–7. 16449747 10.1177/0898010105280112

[12] Braun C, Stangler T, Narveson J, Pettingell S. Animal-assisted therapy as a pain relief intervention for children. Complement Ther Clin Pract. 2009;15(2):105–9. 19341990 10.1016/j.ctcp.2009.02.008

[13] Gagnon J, Bouchard F, Landry M, et al. Implementing a hospital-based animal therapy program for children with cancer: A descriptive study. Can Oncol Nurs J. 2004;14(4):217–22. 15635895 10.5737/1181912x144217222

[14] Wretman C, Risberg A, Grönlund H, Edner A. Negligible allergen presence in hospital dogs after washing. Acta Paediatr. 2025;0:1–7. 10.1111/apa.70112. 10.1111/apa.70112 PMC12336952 40265220

[15] Henry JP. Biological basis of the stress response. Integr Physiol Behav Sci Off J Pavlov Soc. 1992;27(1):66–83. 10.1007/BF02691093 1576090

[16] Uvnäs-Moberg K. Oxytocin may mediate the benefits of positive social interaction and emotions. Psychoneuroendocrinology. 1998;23(8):819–35. 10.1016/s0306-4530(98)00056-0. PMID: 9924739. 9924739 10.1016/s0306-4530(98)00056-0

[17] Gimpl G, Fahrenholz F. The Oxytocin receptor system: structure, function, and regulation. Physiol Rev. 2001;81(2):629–83. 11274341 10.1152/physrev.2001.81.2.629

[18] Petersson M, Diaz-Cabiale Z, Angel Narváez J, Fuxe K, Uvnäs-Moberg K. Oxytocin increases the density of high affinity α2-adrenoceptors within the hypothalamus, the amygdala and the nucleus of the solitary tract in ovariectomized rats. Brain Res. 2005;1049(2):234–9. 15967417 10.1016/j.brainres.2005.05.034

[19] Petersson M, Uvnäs-Moberg K, Nilsson A, Gustafson LL, Hydbring-Sandberg E, Handlin L. Oxytocin and cortisol levels in dog owners and their dogs are associated with behavioral patterns: an exploratory study. Front Psychol. 2017;8:1796. 10.3389/fpsyg.2017.01796. PMID: 29081760; PMCID: PMC5645535. 29081760 10.3389/fpsyg.2017.01796 PMC5645535

[20] Feldman R, Gordon I, Zagoory-Sharon O. Maternal and paternal plasma, salivary, and urinary oxytocin, and parent-infant synchrony: considering stress and affiliation components of human bonding. Dev Sci. 2011;14(4):752–61. 10.1111/j.1467-7687.2010.01021.x. Epub 2010 Dec 16. PMID: 21676095. 21676095 10.1111/j.1467-7687.2010.01021.x

[21] Forsling M, Montgomery H, Halpin D, Windle R, Treacher D. Daily patterns of secretion of neurohypophysial hormones in man: effect of age. Exp Physiol. 1998;83(3):409–18. 9639350 10.1113/expphysiol.1998.sp004124

[22] Amico JA, Ulbrecht JS, Robinson AG. Clearance studies of Oxytocin in humans using radioimmunoassay measurements of the hormone in plasma and urine. J Clin Endocrinol Metab. 1987;64(2):340–5. 3793853 10.1210/jcem-64-2-340

[23] Carter SC, Pournajafi-Nazarloo H, Kramer KM, et al. Oxytocin: behavioral associations and potential as a salivary biomarker. Ann N Y Acad Sci. 2007;1098(1):312–22. 17435137 10.1196/annals.1384.006

